# Adaptive Local–Global Synergistic Perception Network for Hydraulic Concrete Surface Defect Detection

**DOI:** 10.3390/s26030923

**Published:** 2026-01-31

**Authors:** Zhangjun Peng, Li Li, Chuanhao Chang, Mingfei Wan, Guoqiang Zheng, Zhiming Yue, Shuai Zhou, Zhigui Liu

**Affiliations:** 1 School of Information and Control Engineering, Southwest University of Science and Technology, Mianyang 621010, China; pzj1@swust.edu.cn (Z.P.); wanmingfei516@gmail.com (M.W.); handsome@mails.swust.edu.cn (S.Z.); 2 School of Computer Science and Technology, Southwest University of Science and Technology, Mianyang 621010, China; ll@swust.edu.cn (L.L.); chuanhao@mails.swust.edu.cn (C.C.); lil4aintsob@mails.swust.edu.cn (G.Z.); yzm_0924@mails.swust.edu.cn (Z.Y.)

**Keywords:** hydraulic concrete, defect detection, structural health monitoring, multi-scale perception, gated feature fusion

## Abstract

Surface defects in hydraulic concrete structures exhibit extreme topological heterogeneity. and are frequently obscured by unstructured environmental noise. Conventional detection models, constrained by fixed-grid convolutions, often fail to effectively capture these irregular geometries or suppress background artifacts. To address these challenges, this study proposes the Adaptive Local–Global Synergistic Perception Network (ALGSP-Net). First, to overcome geometric constraints, the Defect-aware Receptive Field Aggregation and Adaptive Dynamic Receptive Field modules are introduced. Instead of rigid sampling, this design adaptively modulates the receptive field to align with defect morphologies, ensuring the precise encapsulation of slender cracks and interlaced spalling. Second, a dual-stream gating fusion strategy is employed to mitigate semantic ambiguity. This mechanism leverages global context to calibrate local feature responses, effectively filtering background interference while enhancing cross-scale alignment. Experimental results on the self-constructed SDD-HCS dataset demonstrate that the method achieves an average Precision of 77.46% and an mAP50 of 72.78% across six defect categories. Comparative analysis confirms that ALGSP-Net outperforms state-of-the-art benchmarks in both accuracy and robustness, providing a reliable solution for the intelligent maintenance of hydraulic infrastructure.

## 1. Introduction

Hydraulic concrete structures serve as critical infrastructure components in water conservancy and hydropower engineering. Due to prolonged service in extreme hydro-geological environments characterized by high-head seepage, high-speed water scouring, and dry-wet/freeze–thaw cycles, these surfaces inevitably suffer from irreversible material degradation [1]. Macroscopically, this degradation manifests as various complex defect morphologies: ranging from cracks reflecting structural stress and shrinkage states, to efflorescence and spalling indicating a loss of material durability; and from corrosion caused by high-speed sediment-laden water flow, to exposed rebar and exposed aggregate, which severely threaten structural load-bearing capacity [2]. These defects not only compromise engineering integrity but also serve as core physical indicators for assessing dam safety. Consequently, achieving high-precision, automated detection of these defects under complex environmental interference remains a common challenge urgently requiring resolution in the field of intelligent operation and maintenance of hydraulic engineering [3].

Although deep learning techniques, represented by Convolutional Neural Networks (CNNs), have demonstrated robust feature extraction capabilities in defect detection scenarios such as industrial manufacturing and transportation facilities, they face significant adaptability bottlenecks when transferred to hydraulic scenarios [4]. This challenge stems primarily from two dimensions:

First, defect morphologies exhibit strong topological heterogeneity and cross-scale characteristics. Unlike regular scratches on industrial parts, hydraulic concrete defects present extreme geometric uncertainty. Cracks often exhibit large aspect ratios and tortuous paths, while spalling and corrosion areas appear as irregular patch-like distributions. Traditional CNNs rely heavily on standard convolution kernels with fixed geometric structures. This rigid grid sampling method struggles to effectively cover slender or irregular defect regions, leading to the loss of high-frequency details or dilution by background pixels during feature extraction, making it difficult to precisely delineate defect boundaries.

Second, unstructured environmental noise induces semantic ambiguity. Hydraulic structure surfaces are often covered with complex water stains, moss vegetation, and calcareous deposits. These background textures share high local visual similarity with actual defects, constituting strong interference sources [5]. Existing detection networks, limited by local receptive fields, often lack the ability to understand global image context, leading to misjudgments. Balancing high sensitivity to fine defects with the effective suppression of false alarms caused by environmental noise via global semantic information represents a core contradiction in current research.

To overcome the aforementioned limitations, this paper proposes a hydraulic concrete surface defect detection method based on an Adaptive Local–Global Synergistic Perception Network (ALGSP-Net). The core academic philosophy of this paper lies in breaking the geometric constraints of traditional convolution kernels, endowing the network with adaptive perception capabilities for defect morphologies, and establishing a dynamic calibration mechanism for global and local features. Specifically, the main contributions of this study are as follows:

(1) Targeting the strong irregularity of defect geometries, the Defect-Aware Receptive Field Aggregation Convolution (DaRFAConv) and Adaptive Dynamic Receptive Field Convolution (ARFConv) modules are proposed. Distinct from methods that rely on unstable geometric offsets, these modules employ a prior-guided weight recalibration strategy. This enables the convolution kernels to adaptively modulate the receptive field to encapsulate slender cracks or interlaced exposed rebar areas, significantly enhancing the network’s feature capture capability for complex topological morphologies.

(2) To address misjudgments caused by environmental noise, a Global–Local Synergistic Receptive Field Aggregation Convolution (GARFConv) module and a Defect-aware Dual-stream Gating Fusion (D2GF) module are proposed. This mechanism utilizes global contextual information to calibrate local feature responses and employs a gating strategy to effectively suppress high-frequency background noise interference, achieving precise semantic alignment and fusion of deep and shallow features.

(3) A “Geometric-Semantic Decoupling” strategy is proposed via the Elastic Geometric Encoding (EG-C3k) and Morphology-Adaptive Recursive Aggregator (MA-C2f) modules. In the backbone, the EG-C3k is deployed to preserve the structural continuity of defects, thereby maximizing geometric recall. Conversely, in the neck, the MA-C2f utilizes an input-driven gating mechanism to actively filter artifacts during upsampling, ensuring high semantic precision across multi-scale representations.

(4) A Surface Defect Dataset for Hydraulic Concrete Structures (SDD-HCS) oriented towards real-world scenarios is constructed. Addressing the issues of single samples and simple backgrounds in existing public datasets, this paper collected and annotated high-resolution image data covering six typical defect types: crack, spalling, efflorescence, exposed rebar, exposed aggregate, and corrosion. Containing high-noise backgrounds and multi-scale defect samples, this dataset fills the gap for domain-specific datasets and provides a reliable data benchmark for validating the proposed method.

## 2. Related Works

### 2.1. Overview of Defect Detection Paradigms

The automated detection of surface defects in hydraulic concrete structures has undergone a paradigm shift from traditional image processing to deep representation learning. This section systematically reviews existing research achievements from three dimensions: the evolution of deep convolutional architectures and multi-class disease recognition, lightweight mechanisms for edge deployment, and hybrid architectures for feature enhancement in complex environments. Furthermore, it dissects the persisting challenges in detecting multi-class defects within current hydraulic scenarios.

### 2.2. Deep Convolutional Architectures

Early research on concrete defect detection primarily relied on Convolutional Neural Networks (CNNs) for image-level classification. Chen et al. [6] proposed the NB-CNN framework, which achieved efficient preliminary screening of cracks by fusing Naive Bayes with CNNs. Modarres et al. [7] introduced the ReLU activation function, validating the robustness of deep features in classifying concrete diseases. To address the poor generalization of single datasets, Gao [8] proposed the concept of “Structural ImageNet,” utilizing VGG-16 to achieve fine-grained classification of concrete spalling. Similarly, Feng et al. [9] constructed a five-category disease dataset for hydroelectric hub scenarios based on Inception-v3. However, these classification-oriented models are limited to determining the existence of defects in an image, lacking the capability for precise spatial localization.

To achieve accurate defect localization, object detection algorithms have evolved rapidly. In the realm of two-stage algorithms, Huang et al. [10] improved the anchor box design of Faster R-CNN specifically for dam damage, enhancing multi-damage detection performance. Conversely, single-stage detectors have demonstrated a superior balance between inference speed and accuracy. Zhang et al. [11] were among the first to apply YOLOv3 (You Only Look Once) to bridge surface damage detection. With the iteration of architectures, Raushan et al. [12] systematically evaluated the performance of YOLOv3 through YOLOv10 under multi-feature backgrounds, revealing that YOLOv4 offers an excellent balance for specific concrete damage localization.

Addressing non-crack diseases unique to hydraulic concrete, Cui et al. [13] realized intelligent identification of concrete erosion damage based on an improved YOLOv3, demonstrating the effectiveness of single-stage models on non-crack defects. Following the proposal of the YOLOR architecture, Sun et al. [14] introduced a unified representation of implicit and explicit knowledge in multi-class defect detection for concrete bridges, significantly improving detection accuracy under complex backgrounds. Furthermore, Li et al. [15] and Nguyen et al. [16] explored multi-scale feature fusion and meta-heuristic optimization strategies for concrete dams and spalling diseases, respectively, further expanding the boundaries of deep learning in multi-class disease recognition.

### 2.3. Lightweight Mechanisms and Edge Deployment

Hydraulic inspections frequently rely on UAVs for inaccessible areas, where limited battery and computational resources restrict the deployment of complex models. Driven by these constraints, model lightweighting has become a critical research hotspot to ensure practical engineering viability. Fu et al. [17] developed real-time detection software integrated with an enhanced YOLO model, validating the algorithmic feasibility in practical engineering applications. Chen et al. [18] designed CD-YOLOv8, which leverages a Spatial Context Pyramid to enhance perception from UAV perspectives, achieving real-time detection at 44 FPS on embedded devices. To compress model size while maintaining high precision, Dong et al. [19] proposed a lightweight method based on Joint Knowledge Distillation, where a teacher network guides a student network, drastically reducing the parameter count of dam crack detection models. Pang et al. [20] proposed a high-performance lightweight model combining detection and tracking, resolving the issue of repetitive disease counting in video streams. Additionally, Falaschetti et al. [21] validated the viability of deploying lightweight CNNs on low-power edge devices.

### 2.4. Hybrid Architectures and Emerging Paradigms

Hydraulic concrete surfaces are frequently characterized by complex interference—such as water stains, moss and highly heterogeneous defect morphologies. Pure CNN architectures often exhibit limitations in modeling long-range dependencies and capturing fine-grained features. Consequently, “CNN + Transformer” hybrid architectures and various attention mechanisms have been adopted by scholars for concrete defect detection.

Li et al. [22] proposed CrackTrNet, and Jiang et al. [23] proposed YOLOv5s-road, both employing CNN-Transformer hybrid architectures to utilize Transformers for capturing global contextual information, effectively resolving the discontinuity problem in detecting slender cracks. To enhance feature fusion capabilities, Wang et al. [24] proposed YOLOv8-CDD, combining BiFPN with Transformer modules, which resulted in significant improvements in multi-scale defect detection. Zou et al. [25] designed the SATH-YOLO model, utilizing a Swin Transformer as the prediction head to strengthen the representation of subtle defects in concrete bridges.

Targeting extreme environments specific to hydraulic engineering, Li et al. [26] developed an image-based detection system for underwater corrosion on stilling basin slabs, employing specific image enhancement algorithms to overcome underwater non-uniform illumination and turbidity, thereby filling the gap in automated corrosion detection. Rgarding feature enhancement, Dong et al. [27] introduced Large Selective Kernel Attention (LSKA), simulating long-range dependencies by decomposing large convolution kernels. Chang et al. [28] innovatively proposed a Fourier-Mixture of Experts (FMOE) strategy, utilizing frequency-domain feature expert modules to capture the high-frequency details of cracks. Furthermore, Jha et al. [29] and He [30] verified the anti-interference capabilities of Vision Transformers (ViT) and saliency detection, respectively, under complex backgrounds. To expand the horizon of defect detection, emerging State Space Models (SSM) such as Mamba are gaining attention for their linear complexity and long-range modeling efficiency [31]. For instance, Chen et al. [32] proposed a Shrinkage Mamba Relation Network that achieves robust fault localization even under zero-faulty data conditions. Although currently prominent in mechanical diagnostics, the potential of SSMs to handle signal heterogeneity offers a promising new direction for analyzing complex hydraulic surfaces.

Despite significant strides in the field of concrete disease detection, applications for large-scale hydraulic structures still face three critical bottlenecks: incomplete coverage of defect categories, conflicts between complex topologies and strong noise, and a lack of adaptive perception. Existing methods predominantly focus on conventional diseases, lacking a systematic integration of hydraulic-specific defects such as efflorescence, exposed rebar, and corrosion. Moreover, they struggle to maintain robust representation of cross-scale defects under strong interference like water stains and calcification, while fixed convolution kernels fail to adapt to extremely irregular defect boundaries. To address these issues, this paper proposes ALGSP-Net. By introducing adaptive dynamic receptive fields and a local–global synergistic perception mechanism, this work aims to overcome the technical bottlenecks preventing precise, multi-class defect detection in complex hydraulic scenarios.

## 3. Proposed Method

### 3.1. Overall Architecture

**Figure 1:**
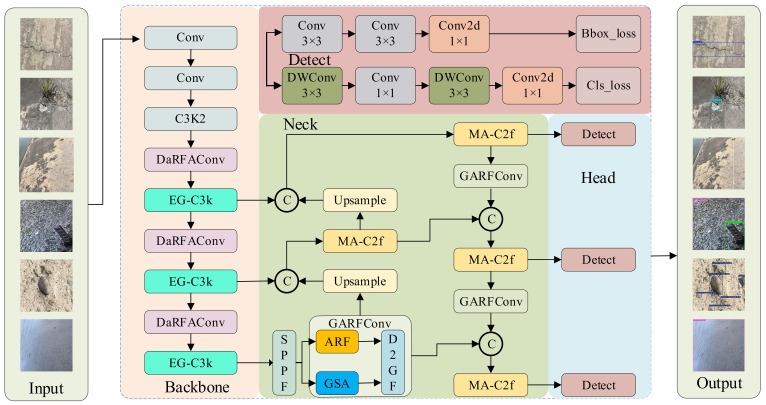
The overall architecture of the proposed adaptive local–global synergistic perception network.

The overall architecture of the proposed ALGSP-Net is illustrated in Figure 1. The model is founded upon the single-stage anchor-free detection framework of YOLO11 [33], retaining its efficient Cross Stage Partial (CSP) gradient flow organization. On this basis, the backbone and neck hierarchies are reconstructed to establish an end-to-end detection system characterized by “elastic perception” and “synergistic calibration”.

Distinct from standard Deformable Convolution Networks (DCNs) [34] which rely on learning physical offsets to shift sampling points—a process often unstable on noisy hydraulic surfaces—we introduce the DaRFAConv and ARFConv modules in the backbone to achieve robust geometric adaptation. As shown in Figure 1, “input” represents the defect image that is input. By employing a prior-guided weight recalibration strategy rather than unconstrained geometric deformation, these modules enable the convolution kernels to adaptively align with irregular morphologies such as cracks and spalling, effectively breaking the constraints of fixed grid sampling without introducing sampling artifacts.

To ensure the preservation of minute defect details during deep-layer downsampling, the fundamental stacking units are upgraded to EG-C3k modules. These units embed adaptive receptive fields and recursive gating mechanisms within dense residual paths, improving upon standard feature propagation blocks by mitigating the semantic gap between deep and shallow layers.

In the neck, a Global-Local Synergistic Feature Pyramid is constructed to address environmental interference. Unlike generic channel attention methods that indiscriminately boost features, the incorporated GARF module (based on the D2GF mechanism) functions as a selective semantic gate. It leverages global context as a baseline to dynamically calibrate local responses, actively filtering unstructured noise like water stains and moss. Subsequently, the MA-C2f module performs a secondary refinement on these aligned features, reinforcing cross-scale semantic consistency. Finally, the synergistically calibrated multi-scale features are fed into a decoupled detection head, where independent classification and regression branches execute category discrimination and bounding box regression for the six typical hydraulic concrete defects, achieving high-precision localization via a dynamic balanced loss. The output shown in Figure 1 represents the prediction results generated by the network.

### 3.2. Defect-Aware Receptive Field Aggregation Convolution

CNNs are inherently constrained by fixed geometric structures and static parameter-sharing mechanisms during feature extraction. This isotropic receptive field mechanism struggles to accommodate non-rigid defect morphologies, particularly when processing defects with slender topological structures or irregular boundaries, often resulting in the loss of critical edge information. Furthermore, complex background textures ubiquitously found in hydraulic environments—such as water stains and moss—exhibit high similarity to specific defects. Standard convolutions, limited by their local receptive fields, frequently fail to effectively discriminate between these structurally analogous features.

**Figure 2:**
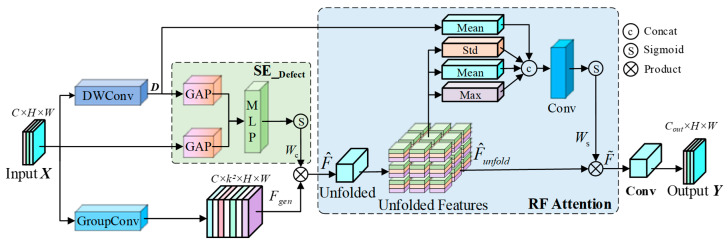
Structure of the defect-aware receptive field aggregation convolution.

To address these limitations, drawing inspiration from [35], we propose the DaRFAConv. As illustrated in Figure 2, this module incorporates an explicit “defect response stream” that serves as a prior guidance signal. Unlike Deformable Convolution Networks (DCNs) [36,37], which physically shift sampling coordinates via learned geometric offsets, DaRFAConv adopts a “soft” feature recalibration strategy. While geometric deformation can be unstable on noisy hydraulic surfaces, our method uses the defect prior to modulate feature importance within a fixed grid. This ensures the network focuses on defect topologies without introducing interpolation artifacts associated with irregular sampling in high-noise environments. By leveraging a dual-view feature recalibration mechanism, DaRFAConv dynamically modulates spatial and channel weights within the receptive field, thereby facilitating the precise capture of irregular defect morphologies.

DaRFAConv decouples the standard convolution operation into two distinct processes: feature unfolding and weighted aggregation, while simultaneously incorporating an explicit defect prior. Given an input feature map $X\in R^{C\times H\times W}$, we first employ a lightweight depthwise convolution (DWConv) to extract the high-frequency defect response map, D. In contrast to standard convolutions that fuse information across channels, DWConv performs spatial filtering on each channel independently. This isolation forces the kernels to capture local intensity gradients rather than semantic combinations. Consequently, the layer acts as a learnable high-pass filter, effectively highlighting high-frequency components such as crack boundaries and texture anomalies:

(1) D=σ(DWConv(X))

where σ denotes a non-linear activation function. Since the boundaries of cracks and spalling typically manifest as high-frequency transitions within an image, D provides a coarse localization of potential defect regions, serving as a structural guide for subsequent attention mechanisms.

In the channel dimension, specific convolution kernels are often susceptible to interference from background noise. To mitigate this bias, we design the SE_Defect module. Unlike the conventional Squeeze-and-Excitation (SE) block, which relies solely on the global statistics of the feature map itself, SE_Defect explicitly leverages the defect response map D to guide channel selection. The original features X and the defect features D are independently subjected to Global Average Pooling (GAP). The resulting vectors are concatenated and processed by a Multilayer Perceptron (MLP) to generate the channel weights $W_{c}$:

(2) $$W_{c}=Sigmoid(MLP([GAP(X),GAP(D)]))$$

Because D encodes explicit spatial information regarding defect locations, the network learns to distinguish between channels responsive to defects and those responsive to background noise. Consequently, background channel activations that misalign with the defect prior are actively suppressed.

To emulate sliding window operations with a large receptive field, we utilize grouped convolutions to generate the unfolded features $F_{gen}\in R^{(C\cdot K^{2})\times H\times W}$, where K represents the convolution kernel size. In our experiments, K is set to 3. This value was empirically selected as it efficiently captures fine-grained local defect features while minimizing the computational redundancy and background noise interference associated with larger kernels. These features are modulated by the channel weights $\hat{F}=F_{gen}⊙W_{c}$, thereby achieving channel-level denoising guided by the defect prior. Through a rearrangement operation, local neighborhood information from the spatial dimensions of $\hat{F}$ is mapped to the channel dimension, resulting in the unfolded feature ${\hat{F}}_{unfold}\in R^{C\times (H\cdot K)\times (W\cdot K)}$. Within this unfolded feature space, identifying which pixels within the K×K receptive field are most critical for aggregation is essential. The DaRFAConv module utilizes not only the intrinsic statistical moments of the features (maximum, mean, and variance) but also incorporates the local response intensity from the defect map as a fourth statistic.

Let $S_{rf}\in R^{4\times (H\cdot K)\times (W\cdot K)}$ denote the receptive field statistical descriptor, computed as follows:

(3) $$S_{rf}=\mathrm{Concat}(\mathrm{Max}({\hat{F}}_{unfold}),\mathrm{Mean}({\hat{F}}_{unfold}),\mathrm{Std}({\hat{F}}_{unfold}),\mathrm{Mean}(|\mathrm{Interp}(D)|))$$

where Std(⋅) computes the local standard deviation to capture texture complexity, and Interp(⋅) represents the interpolation operation used to align the defect features with the unfolded dimensions. Subsequently, a shared-weight convolutional layer is utilized to generate a spatial attention map, performing pixel-wise weighting on the unfolded features:

(4) $$\tilde{F}={\hat{F}}_{unfold}⊙\mathrm{Sigmoid}({\mathrm{Conv}}_{1\times 1}(S_{rf}))$$

This mechanism empowers the convolution kernel to adaptively deform according to the local geometric characteristics of the defect, concentrating weights on valid regions while attenuating background noise. Finally, the features, having undergone dual recalibration, are aggregated and dimensionally reduced via a standard convolutional layer to restore the target output dimensions.

### 3.3. Adaptive Dynamic Receptive Field Convolution

**Figure 3:**
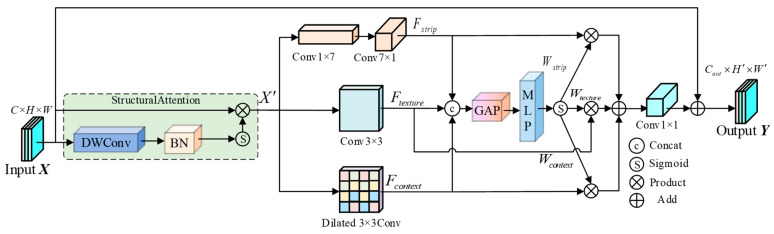
Structure of the adaptive dynamic receptive field convolution.

To address the high geometric heterogeneity of multi-type hydraulic concrete defects and the statistical similarity of mimetic noise, we propose the ARFConv module. Building upon the “defect prior-driven” philosophy of DaRFAConv, ARFConv further refines feature modeling through a multi-branch architecture, as illustrated in Figure 3. This module explicitly introduces three complementary branches within the same layer to capture strip-like structures, texture details, and contextual semantics, respectively. By employing an input-adaptive gating mechanism for dynamic fusion, ARFConv realizes a “decomposable morphology, complementary information, and adaptive fusion” modeling paradigm, thereby generating highly discriminative defect representations.

As illustrated in Figure 3, given an input feature map $X\in R^{C\times H\times W}$, ARFConv adopts an “Enhance-Extract-Fuse-Residual” design paradigm to generate the output feature map $Y\in R^{C_{out}\times H^{\prime }\times W^{\prime }}$. To prevent weak defect signals from being overwhelmed by background noise within subsequent parallel branches, we introduce a lightweight Structural Attention module prior to feature extraction. This module is specifically engineered to accentuate spatial structural cues, particularly in regions sensitive to defect boundaries and texture variations. This enhancement process is defined as:

(5) $$X^{\prime }=X⊙\sigma (BN(DWConv_{3\times 3}(X)))$$

where $DWConv_{3\times 3}$ denotes a 3×3 Depthwise Convolution, BN represents Batch Normalization, σ corresponds to the Sigmoid activation function, and ⊙ indicates element-wise multiplication, $X^{\prime }$ denotes the feature map with enhanced structural representation.

The efficacy of ARFConv stems from its three parallel feature extraction branches, each tailored to specific morphological characteristics of hydraulic concrete defects. We decouple the spatial feature extraction space into three orthogonal transformation operators:

(1) To address the slender topology inherent to defects like cracks, we employ Strip Convolution (StripConv) [38]. This operator decomposes the standard two-dimensional convolution into two sequential group convolutions of size 1×k and k×1. This factorization constructs an elongated receptive field, maximizing the capture of long-range linear dependencies along the crack trajectory:

(6) $$F_{strip}=\delta (BN(Conv_{k\times 1}(Conv_{1\times k}(X^{\prime }))))$$

where k=7 and δ denotes the ReLU activation function. Low-rank decomposition allows the receptive field to extend axially, enhancing continuity response along the crack path.

(2) Texture Branch: We utilize a standard 3×3 Depthwise Convolution to extract local, high-frequency texture information, defined as:

(7) $$F_{texture}=\delta (BN(DWConv_{3\times 3}(X)))$$

(3) Context Branch: To capture broader contextual semantics—crucial for identifying large-area defects without increasing parameter overhead—we employ dilated convolution with a dilation rate of 3:

(8) $$F_{context}=\delta (BN({\mathrm{Conv}}_{3\times 3,d=3}(X^{\prime })))$$

To achieve adaptive receptive field modulation, we engineer a dynamic weight generator that assigns importance to each branch based on input content. The outputs of the three branches are concatenated along the channel dimension to form the aggregated feature $F_{cat}=[F_{strip},F_{texture},F_{context}]\in R^{3C\times H^{\prime }\times W^{\prime }}$. Leveraging GAP to embed global spatial information into a 1×1 vector, we employ a bottleneck structure consisting of two 1×1 convolutional layers. This design efficiently models the nonlinear inter-channel correlations—acting effectively as a MLP to generate the dynamic attention vector W:

(9) $$W=\sigma (CONV_{1\times 1}^{expand}(\delta (CONV_{1\times 1}^{reduce}(GAP(F_{cat})))))$$

where σ represents the Sigmoid function, and δ denotes the ReLU activation function. The generated weight vector $W\in R^{3C\times 1\times 1}$ is split into $W_{strip},W_{texture},W_{context}\in R^{C\times 1\times 1}$. These dynamic weights modulate their corresponding branch features via weighted fusion:

(10) $$F_{fused}=F_{strip}⊙W_{strip}+F_{texture}⊙W_{texture}+F_{context}⊙W_{context}$$

This process enables ARFConv to adaptively select the optimal feature combination. The fused feature $F_{fused}$ undergoes channel projection via a 1×1 convolution layer to match the output dimension $C_{out}$. To facilitate gradient propagation and preserve primitive information, a residual connection is introduced. An identity mapping is used if input/output dimensions and spatial resolutions align; otherwise, a convolution performs the necessary alignment. By integrating morphology-aware multi-branch structures with dynamic feature fusion, ARFConv serves as a high-efficiency feature extraction unit for hydraulic concrete surface defect detection.

### 3.4. Global-Local Synergistic Receptive Field Aggregation Module

In the preceding sections, we introduced DaRFAConv (Section 3.2) to enhance signal saliency amidst background noise and ARFConv (Section 3.3) to accommodate the geometric heterogeneity of defects. While these modules effectively modulate receptive fields through dynamic adjustment and multi-scale expansion, they remain inherently constrained to local neighborhoods. In complex hydraulic concrete environments, relying solely on local receptive fields proves insufficient for resolving semantic ambiguity—where background textures mimic defect features. To address this, we propose the Global-Local Synergistic Receptive Field Aggregation (GARFConv) module. This architecture establishes an orthogonal, complementary dual-branch system: it retains the robust local morphological fitting capability of ARFConv while simultaneously employing an improved global self-attention mechanism within a global branch. By establishing full-image dependencies, this design leverages macroscopic semantic information to calibrate local feature responses, effectively distinguishing true defects from environmental noise.

**Figure 4:**
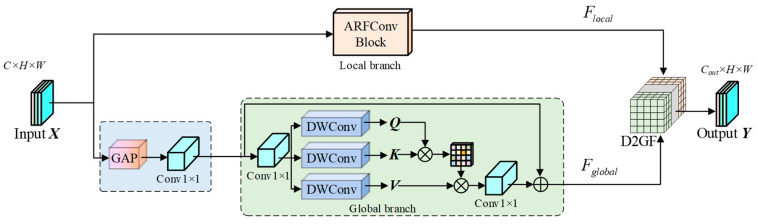
Structure of the global-local synergistic receptive field aggregation convolution module.

As illustrated in Figure 4, to capture global contextual information within a constrained computational budget, we engineer a high-efficiency Global Self-Attention Branch. Given an input feature map $X\in R^{C\times H\times W}$, we first align spatial dimensions via adaptive average pooling to obtain $X_{aligned}$ if scale discrepancies exist.

Standard self-attention mechanisms inherently lack positional awareness, typically necessitating explicit positional encodings. To internalize this capability and reinforce local feature extraction, we embed a Local Inductive Bias directly into the projection phase of the Query (Q), Key (K), and Value (V). Prior to establishing global correlations, features undergo a 1×1 convolutional mapping followed immediately by a 3×3 Depthwise Convolution:

(11) $$Q,K,V={\mathrm{Split}(\mathrm{DWConv}}_{3\times 3}{(\mathrm{Conv}}_{1\times 1}(X_{aligned})))$$

where ${\mathrm{Conv}}_{1\times 1}$ facilitates channel mixing, ${\mathrm{DWConv}}_{3\times 3}$ is responsible for the injection of spatial local information. To compute global correlations, we flatten the spatial dimensions H and W into a single dimension N=H×W, reshaping the features into query, key, and value matrices:

(12) $$Q,K,V\in R^{B\times h\times (C/h)\times N}$$

where h denotes the number of attention heads. We employ a dot-product attention mechanism, introducing a learnable temperature parameter $\tau \in R^{h\times 1\times 1}$ to dynamically modulate the sharpness of the softmax distribution. This yields the global attention map:

(13) $$A=\mathrm{Softmax}(\frac{\mathrm{Norm}(Q)\cdot \mathrm{Norm}{\left(K\right)}^{⊤}}{\tau })$$

where Norm(⋅) represents L2 normalization along the channel dimension, utilized to stabilize gradients during the training process. The final global feature representation, $F_{global}$, is obtained by aggregating the value vectors via the attention map and re-projecting them back to the original spatial dimensions:

(14) $$F_{global}={\mathrm{Conv}}_{project}(A\cdot V)$$

In regions characterized by complex backgrounds, the network automatically lowers τ to smooth the attention distribution. This leverages broader contextual information to suppress noise, enabling the model to capture dependencies between any two arbitrary points in the image and establishing a full-scope receptive field.

The local branch of GARFConv employs the ARFConv module defined in Section 3.3. This branch utilizes its multi-morphology convolution kernels to precisely delineate the local boundaries and texture details of defects, with its output denoted as $F_{local}=F_{ARF}(X_{in})$.

Local features provide precise positional cues, whereas global features offer macroscopic semantic context. After obtaining the local geometric features $F_{local}$ and the global semantic features $F_{global}$, we avoid naive addition or concatenation. Instead, integration is achieved through the D2GF module designed in Section 3.5:

(15) $$Y=D2GF([F_{local},F_{global}])$$

By incorporating D2GF, we ensure that every receptive field aggregation operation undergoes rigorous semantic and geometric alignment, thereby providing highly robust feature representations for the subsequent detection head.

### 3.5. Defect-Aware Dual-Stream Gating Fusion Module

**Figure 5:**
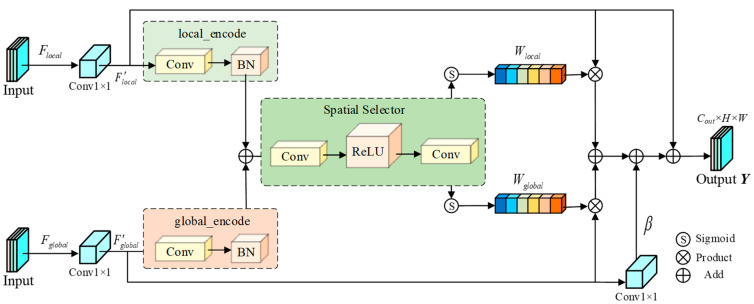
Structure of the defect-aware dual-stream gating fusion module.

To efficiently integrate the complementary local view features from the ARFConv module with the global semantic features, we propose the D2GF. By employing a pixel-level dynamic routing gating mechanism, this module achieves a dynamic equilibrium between preserving local texture fidelity and leveraging global semantic guidance. Furthermore, recognizing the risk that a single gating mechanism may fail under extreme background interference, we introduce an independent spatial gating strategy alongside a global semantic bypass. This design significantly enhances the robustness and efficacy of feature fusion, as illustrated in Figure 5.

The D2GF module ingests a pair of feature maps: the local morphological feature $F_{local}\in R^{C_{l}\times H\times W}$ derived from the local branch, and the global semantic feature $F_{global}\in R^{C_{g}\times H\times W}$ from the global branch. To standardize feature dimensionality, we employ 1×1 convolutional layers to project both inputs into a unified channel space $C_{dim}=\min(C_{l},C_{g})$, yielding the aligned features ${F^{\prime }}_{local}$ and ${F^{\prime }}_{global}$. To generate the subsequent spatial gating weights, we utilize lightweight, bias-free 1×1 convolutions to encode the aligned features, extracting representations optimized for spatial selection:

(16) $$F_{l}={\mathrm{Conv}}_{enc\_l}({F^{\prime }}_{local}),\;F_{g}={\mathrm{Conv}}_{enc\_g}({F^{\prime }}_{global})$$

To achieve pixel-level adaptive fusion, D2GF adopts a content-based Independent Spatial Gating mechanism. Unlike rigid zero-sum games (e.g., Softmax), this design permits local and global features to maintain high activation states simultaneously within specific regions, thereby maximizing information retention. The encoded features are aggregated via $F_{agg}=F_{l}+F_{g}$ and subsequently fed into a spatial selector to decode two independent confidence maps. These maps undergo independent Sigmoid activation to generate mutually unconstrained spatial weights $W_{local},W_{global}\in {\left[0,1\right]}^{H\times W}$:

(17) $$\left[L_{local},L_{global}]={\mathrm{Conv}}_{select}(\mathrm{ReLU}({\mathrm{Conv}}_{reduce}(F_{agg}))\right)$$

(18) $$W_{local}=\sigma (L_{local}),\;W_{global}=\sigma (L_{global})$$

These weights spatially modulate the original aligned features, which are then summed to produce the initial fused representation. A standard convolution block equipped with Batch Normalization (BN) and SiLU activation performs further integration:

(19) $$Y_{fused}=\mathrm{Proj}({F^{\prime }}_{local}⊙W_{local}+{F^{\prime }}_{global}⊙W_{global})$$

Although the gating mechanism filters features effectively, local features often exhibit stronger gradient responses during backpropagation in deep networks. This disparity can lead to “Semantic Degradation,” where the global branch is progressively suppressed. To mitigate this risk, we construct a Global Semantic Bypass alongside the weighted fusion path. Even if local noise forces strong suppression of spatial gates $W_{local}$ and $W_{global}$, this bypass ensures that global contextual information stably guides the final representation. The bypass applies a lightweight transformation to the aligned global feature ${F^{\prime }}_{global}$, which is then superimposed onto the fused feature via a learnable scaling coefficient β:

(20) $$Y_{enhanced}=Y_{fused}+\beta \cdot {\mathrm{Conv}}_{bypass}({F^{\prime }}_{global})$$

The introduction of β allows the network to automatically regulate the contribution of global bottom-line information based on training dynamics. Finally, to maintain precise localization capabilities for defect boundaries and facilitate gradient propagation, the output of D2GF adopts a residual connection form anchored by the local feature:

(21) $$Y_{out}={F^{\prime }}_{local}+Y_{enhanced}$$

The D2GF module realizes an efficient and robust fusion of local details and global semantics, providing high-quality multi-scale feature representations.

### 3.6. Morphology-Adaptive Encoding and Recursive Gating Mechanism

In ALGSP-Net, we devise a “Geometric-Semantic Decoupling” optimization strategy. Specifically, we deploy the EG-C3k within the deep feature extraction backbone to maximize the topological recall of defects. Conversely, we station the MA-C2F within the feature fusion neck to ensure the semantic precision of the representations.

#### 3.6.1. Elastic Geometric Encoding Based on EG-C3k

**Figure 6:**
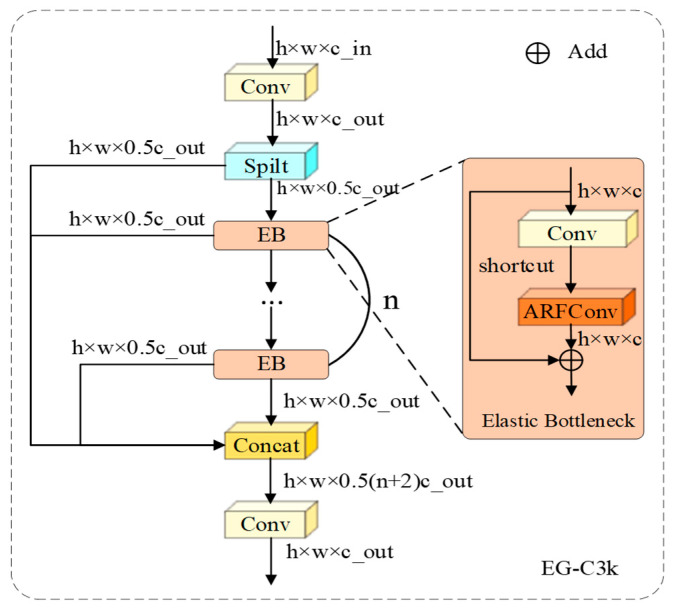
Structure of the Elastic Geometric Encoding module.

In the deep stages of the Backbone network, the core objective of feature extraction is to endow the network with topological invariance. This requires accurately tracking and preserving the boundary trajectories of defects during downsampling. While the traditional C3k2 module of the YOLO series optimizes gradient flow via the Cross Stage Partial (CSP) architecture, its internal stacking of standard convolution kernels is constrained by fixed orthogonal sampling grids. This rigidity often leads to Feature Fragmentation when processing slender or curved defects. To mitigate this, we construct the EG-C3k, with its structure illustrated in Figure 6. This module retains the dense gradient path design of CSP but reconstructs the internal bottleneck unit into an Elastic Bottleneck. This is achieved by replacing the standard 3×3 convolution with ARFConv:

(22) $$T_{bottle}=T_{ARF}(T_{Conv}(X_{in}))+X_{in}$$

where $T_{conv}$ denotes the spatial convolution used for channel adjustment, while $T_{ARF}$ integrates adaptive offset prediction with the multi-view feature decoupling mechanism. This design deeply embeds “elastic deformation” capabilities into every residual path of the backbone network. For cracks, the receptive field dynamically stretches axially, ensuring continuous feature transmission along the topological structure. For minute defects, the receptive field contracts toward the centroid, minimizing the intrusion of background pixels. By stacking EG-C3k units, the network establishes shape-aware representations that are highly robust to irregular defects prior to multi-scale fusion, effectively preventing the loss of geometric details within deep semantic layers.

#### 3.6.2. Recursive Gating Aggregation Based on MA-C2f

**Figure 7:**
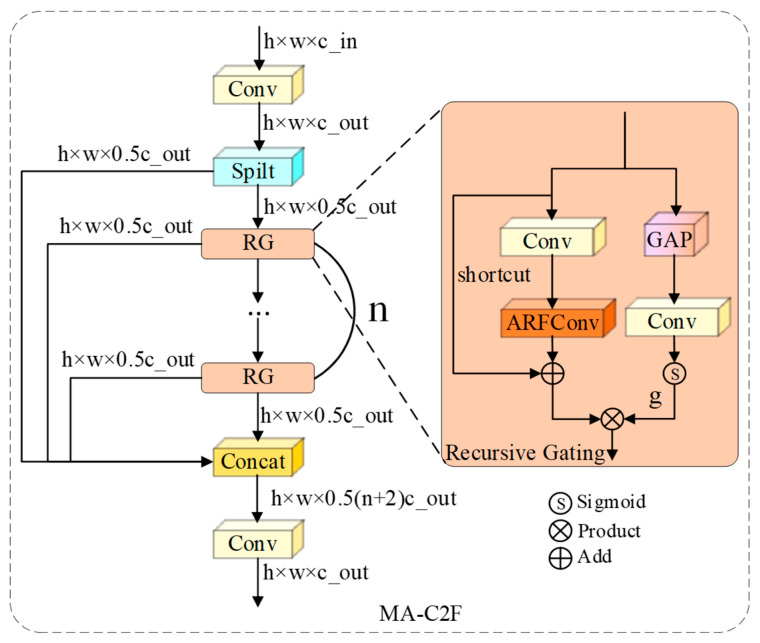
Structure of the Morphology-Adaptive Recursive Aggregator module.

Upsampling operations within the feature pyramid fusion stage frequently propagate unstructured background noise from shallow layers into deep representations, precipitating the generation of spurious artifacts. Drawing inspiration from the Area Attention paradigm of YOLO12 [39] and tailoring it to the specific nuances of hydraulic scenarios, we propose the MA-C2f module. The architecture of this module is depicted in Figure 7.

MA-C2f incorporates an Input-Driven Recursive Gating strategy. Let the feature list resulting from the CSP split be denoted as $Y=[y_{1},y_{2},\ldots ,y_{n}]$. For each processing unit within this sequence, the feature update rule transcends simple convolutional transformation, evolving into a dynamic modulation integrated with global context. Specifically, for the k bottleneck layer with input $y_{k-1}$, a lightweight global perception branch first generates a prior descriptor g:

(23) $$g=\sigma ({\mathrm{Conv}}_{1\times 1}(\mathrm{GAP}(y_{k-1})))$$

where GAP represents Global Average Pooling and σ denotes the Sigmoid activation function. This descriptor serves to gate the features processed by ARFConv:

(24) $$y_{k}=T_{ARF}(y_{k-1})⊙g$$

This architecture establishes a critical “Look-Before-Process” mechanism, where the network evaluates the Input Context to determine the necessity of retaining processing results.

If the global statistical characteristics of the input $y_{k-1}$ suggest a non-defect background, the gating signal g converges towards 0, actively suppressing the activation of the subsequent ARF convolution. Since this operation is executed recursively at each branch node of the CSP, noise is progressively filtered layer-by-layer during the cascading process, thereby ensuring the Semantic Purity of the final aggregated features.

The coordinated deployment of these two modules is pivotal to the architecture of ALGSP-Net. EG-C3k concentrates on maximizing Geometric Recall; through the elastic deformation capabilities of ARFConv, it ensures that even minute, inconspicuous defects are fully encoded, effectively resolving the problem of missed detections. Conversely, MA-C2f prioritizes maximizing Semantic Precision; through its recursive gating strategy, it rigorously filters background artifacts introduced during upsampling, effectively resolving the problem of false alarms.

### 3.7. Loss Function

To facilitate robust multi-class defect detection for hydraulic concrete surfaces, we formulate a composite objective function, $L_{\mathrm{total}}$. Considering the extreme variability of defect features and the inherent boundary ambiguity of corrosion-type defects, our hybrid objective combines Focaler-CIoU loss [40], Distribution Focal Loss (DFL) [41], and Binary Cross Entropy (BCE) loss [42]. The total loss is defined as:

(25) $$L_{total}=\lambda_{box}L_{Focaler-CIoU}+\lambda_{dfl}L_{DFL}+\lambda_{cls}L_{BCE}$$

where $\lambda_{box}$, $\lambda_{dfl}$, and $\lambda_{cls}$ are hyperparameters balancing the contributions of bounding box regression, distribution learning, and classification tasks, respectively.

Hydraulic concrete defects exhibit dual characteristics: geometric anisotropy and spatial uncertainty. We address these challenges through a dual-component regression loss strategy.

(1) Localization Loss: Standard IoU loss gradients are typically dominated by simple samples, limiting convergence on difficult cases. To prioritize hard samples, we employ Focaler-CIoU. This method reshapes the IoU loss curve via a linear interval mapping [d,u], thereby amplifying the gradient contribution of hard detection samples. Combined with the geometric constraints of CIoU (overlap, distance, and aspect ratio), it is defined as:

(26) $$L_{Focaler-CIoU}=1-IoU^{focaler}+\frac{\rho^{2}(b,b^{gt})}{c^{2}}+\alpha v$$

(27) $$IoU^{focaler}=\left\{\begin{array}{ll} 0, & IoU<d\\ \frac{IoU-d}{u-d}, & d\leq IoU<u\\ 1, & IoU\geq u \end{array}\right.$$

where d=0.0 and u=0.95 serve as focusing thresholds. This mechanism significantly improves the localization precision for slender, anisotropic defects by re-weighting the loss contribution based on overlap quality.

(2) Uncertainty-Aware Regression: DFL Unlike cracks which possess distinct edges, defects such as efflorescence and corrosion exhibit gradual transitions from damaged to intact concrete, rendering boundaries blurred and annotations inherently uncertain. Standard regression methods assume a Dirac δ distribution, which fails to model this ambiguity. We introduce DFL to model bounding box offsets as a general probability distribution. Given a true label y and its two nearest discrete bin values $y_{i}$ and $y_{i+1}$ (where $y_{i}\leq y\leq y_{i+1}$), DFL optimizes the predicted probabilities $S_{i}$ and $S_{i+1}$ to concentrate mass near the true location:

(28) $$L_{DFL}(S_{i},S_{i+1})=-\left(\left(y_{i+1}-y)\log(S_{i})+(y-y_{i})\log(S_{i+1}\right)\right)$$

By learning the probability density of boundary locations, DFL enables the network to capture the spatial uncertainty inherent to eroded surfaces, achieving superior detection performance on morphologically irregular defects.

(3) Classification Loss: To achieve precise identification across multiple hydraulic concrete defect categories, we employ Binary Cross Entropy as the classification loss function. This strategy decouples the multi-class task into independent binary classification problems, allowing the network to predict the presence probability of each category independently—a flexibility crucial for complex defect scenarios where classes may co-occur.

The BCE loss computes the divergence between the predicted value and the ground truth label via the Sigmoid activation function:

(29) $$L_{cls}=L_{BCE}=-\sum_{n=1}^{N}\left[y_{n}\log(p_{n})+(1-y_{n})\log(1-p_{n})\right]$$

where N represents the total number of defect categories, $y_{n}\in \{0,1\}$ denotes the ground truth label for the n-th class, and $p_{n}$ represents the predicted probability of the n-th class after Sigmoid activation.

## 4. Experiments and Discussion

### 4.1. Datasets

To rigorously evaluate the defect detection performance of ALGSP-Net under complex real-world operating conditions and to verify its cross-domain generalization capabilities, we adopted a dual-verification strategy comprising a “self-constructed specialized dataset” and a “public benchmark dataset.” The primary experimental data were derived from our proprietary Surface Defect Dataset for Hydraulic Concrete Structures (SDD-HCS), supplemented by a publicly available dataset on general construction concrete defects.

Self-Constructed SDD-HCS Dataset: Raw imagery for the SDD-HCS was acquired from multiple hydraulic concrete infrastructures located in Southwest China. Addressing the challenges of inaccessibility and the significant height of these structures, we employed an industrial-grade DJI Matrice 350 RTK unmanned aerial vehicle (UAV) (DJI, Shenzhen, China) equipped with a Zenmuse H30 high-definition payload camera (DJI, Shenzhen, China). Close-range photogrammetry was conducted at distances of 2–5 m from the target surfaces. Standardized camera settings were maintained during acquisition to ensure consistent exposure under varying illumination, with ISO set between 100 and 400, aperture ranging from f/2.8 to f/5.6, and shutter speeds of 1/500–1/1000 s. Unlike video-based collection, images were acquired via discrete photography. Flight paths were planned to maintain distinct fields of view with minimal spatial overlap between adjacent capture points, preventing data leakage caused by inter-frame redundancy. This process yielded 2835 raw inspection images with a resolution of 3840×2160 pixels. A manual screening process was subsequently applied to remove any accidentally overlapping or visually similar images, ensuring spatial independence.

**Figure 8:**
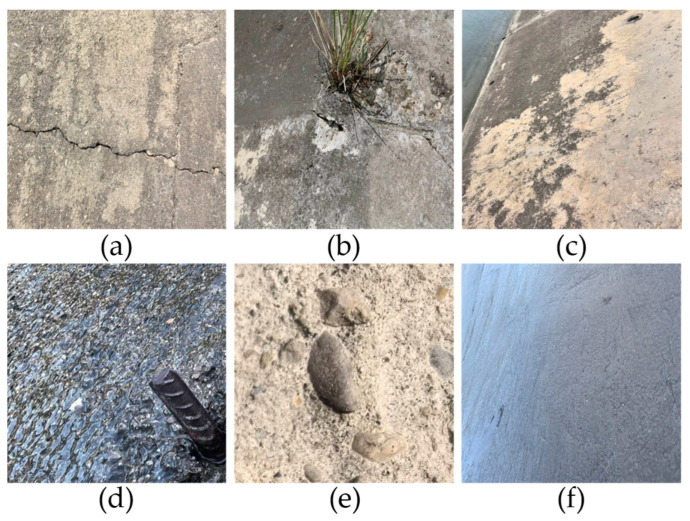
Representative samples from the SDD-HCS dataset. (**a**) Crack; (**b**) Spalling; (**c**) Efflorescence; (**d**) Exposed rebar; (**e**) Exposed aggregate; (**f**) Corrosion.

For network compatibility, the raw images were cropped into non-overlapping patches of 640×640 pixels. After filtering background-only samples, the final dataset contained 7289 images. Annotations were performed using Labelme (v5.4.0) software for six defect types: crack, spalling, efflorescence, corrosion, exposed rebar, and exposed aggregate. Based on the strict spatial independence established during acquisition, the dataset was partitioned into training (5831), validation (729), and testing (729) sets using stratified random sampling with an 8:1:1 ratio. Representative samples and detailed shooting conditions are presented in Figure 8.

**Figure 9:**
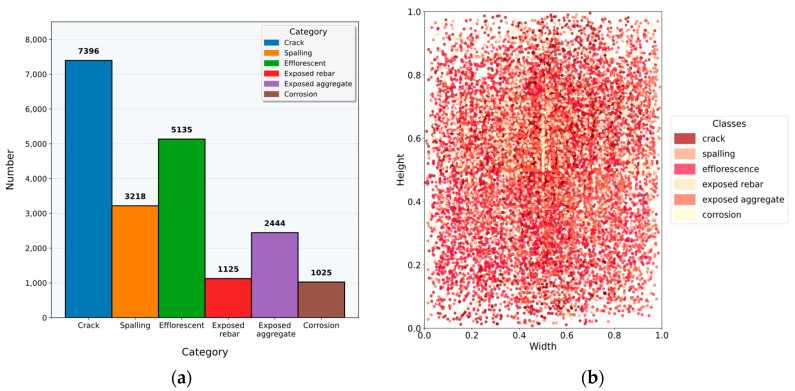
Statistical analysis of the SDD-HCS dataset. (**a**) Distribution of instance counts per category; (**b**) Normalized distribution of defect bounding box dimensions.

Figure 9 provides a detailed quantitative analysis of the distributional properties of the SDD-HCS dataset, exposing two intrinsic challenges inherent to this domain: severe class imbalance and drastic intra-class scale variation. As illustrated in Figure 9a, the instance frequency across categories exhibits a pronounced long-tailed distribution. Specifically, while crack instances are abundant (N=7396), categories such as exposed rebar and corrosion are significantly underrepresented, containing only 1125 and 1025 instances, respectively. This profound disparity necessitates a detection architecture with exceptional robustness, capable of extracting discriminative semantic representations even from sparse supervision signals. Furthermore, Figure 9b illustrates the distribution of bounding box dimensions, normalized relative to the image size. The plot highlights significant geometric differences across categories, as well as substantial scale variations within individual classes. These distributional characteristics underscore the necessity of integrating local detail capture with global contextual perception, validating the design rationale of the proposed network for complex hydraulic concrete environments.

To validate the generalization capability of ALGSP-Net within generic construction concrete environments, we incorporated a publicly accessible multi-class defect dataset [43]. This dataset comprises 7363 images standardized to a resolution of 640×640 pixels and encompasses six distinct defect categories: exposed rebar, corrosion, cracks, spalling, efflorescence, and delamination. Integrating this external benchmark allows for a rigorous assessment of the model’s robustness in non-hydraulic-specific scenarios.

### 4.2. Experimental Environment

To ensure experimental reproducibility and computational efficiency, all training and inference procedures were conducted on a unified workstation environment. The hardware infrastructure is equipped with an NVIDIA GeForce RTX 4090 GPU (24 GB VRAM) and an Intel Core i9-14900K processor, supported by 64 GB of RAM running on the Ubuntu 24.04 operating system. The software stack includes CUDA 12.4, Python 3.12, and the PyTorch 2.4.1 deep learning framework. Detailed hardware specifications are enumerated in Table 1.

Table 2 lists the critical hyperparameters employed during the training process. These values were determined through rigorous preliminary experiments to optimize both model convergence speed and detection accuracy.

### 4.3. Evaluation Metrics

To objectively quantify the comprehensive performance of ALGSP-Net in detecting surface defects on hydraulic concrete, this study adheres to the Standard Evaluation Protocol prevalent in object detection. Based on the Intersection over Union (IoU) between predicted bounding boxes and Ground Truth (GT) [44], detection results are categorized into True Positives (TP), False Positives (FP), and False Negatives (FN). Upon this foundation, we construct a multidimensional evaluation framework comprising Precision, Recall, Average Precision (AP), and F1-Score.

Precision (P) characterizes the model’s capability to resist interference from unstructured background noise, such as water stains and moss. Conversely, Recall (R) reflects the model’s sensitivity in capturing difficult targets, including minute cracks and early-stage corrosion. These metrics are defined as follows:

(30) $$P=\frac{TP}{TP+FP}$$

(31) $$R=\frac{TP}{TP+FN}$$

Average Precision (AP) is employed to measure the detection quality for a single category. Mathematically, AP is defined as the integral area under the Precision-Recall (P-R) curve. Physically, it represents the average prediction quality of the model for a specific class across varying recall levels:

(32) $$AP=\int_{0}^{1}P(R)dR$$

To evaluate global detection performance across multiple categories (in this study, K=6), we compute the mean of AP values for all classes, denoted as mean Average Precision (mAP). This metric serves as the standard for measuring the overall robustness of the detector. Our experiments specifically focus on mAP50, mAP75, and mAP50-95:

(33) $$mAP=\frac{1}{K}\sum_{i=1}^{K}AP_{i}$$

The composite metric F1-Score, calculated as the harmonic mean of Precision and Recall, is utilized to evaluate the model’s comprehensive stability under a single threshold:

(34) $$F1=2\cdot \frac{P\cdot R}{P+R}$$

In addition to accuracy metrics, we evaluate model efficiency using Parameters (Params) to quantify spatial complexity and memory footprint, and Giga Floating-point Operations (GFLOPs) to assess computational cost. Specifically, the reported GFLOPs measure the computational load during the inference phase (forward pass), benchmarked at a standardized input resolution of 640×640 pixels.

### 4.4. Results and Analysis

#### 4.4.1. Performance Evaluation on the SDD-HCS Dataset

**Figure 10:**
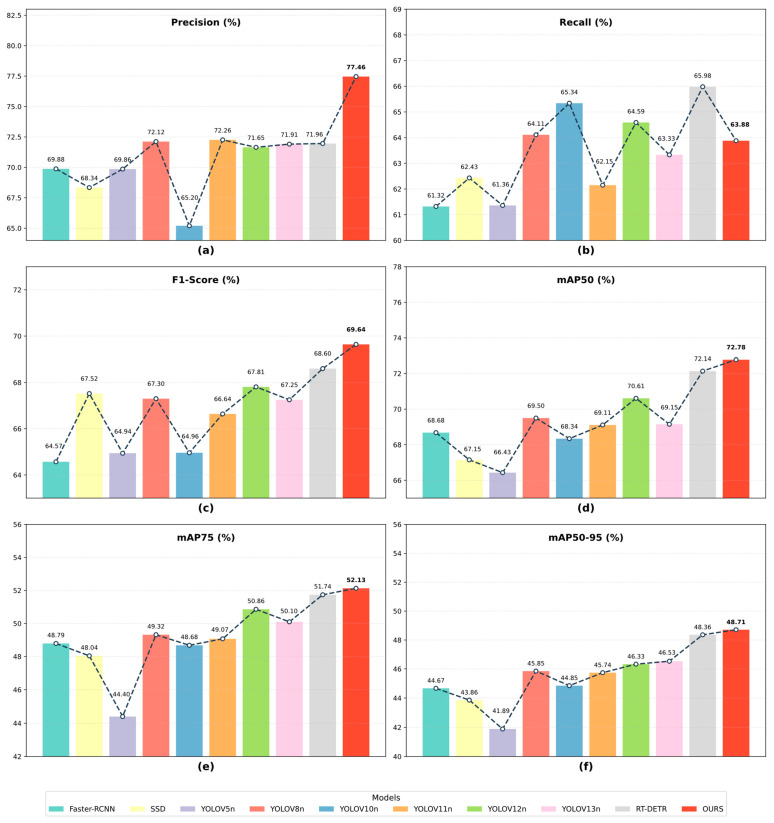
Comparative performance analysis of various detection models on the SDD-HCS dataset. The dashed lines connect the metric values to visualize the performance variation across different models. (**a**) Comparison of Precision; (**b**) Comparison of Recall; (**c**) Comparison of F1-Score; (**d**) Comparison of mAP50; (**e**) Comparison of mAP75; (**f**) Comparison of mAP50-95.

To rigorously evaluate the holistic efficacy of ALGSP-Net in detecting hydraulic concrete surface defects, we conducted a comparative benchmarking study on the SDD-HCS dataset against nine representative state-of-the-art methods. The baseline models encompass the two-stage Faster R-CNN [45], the one-stage SSD [46], various iterations of the YOLO series such as YOLOv5n [47], YOLOv8n [48], YOLOv10n [49], YOLOv11n [33], YOLOv12n [39], and YOLOv13n [50], and the Transformer-based RT-DETR [51]. To ensure experimental fairness, all models were trained and evaluated using identical data partitions and consistent hyperparameter configurations. Table 3 details the quantitative performance metrics, while Figure 10 visually illustrates the comparative landscape across key performance dimensions.

Evaluation of Detection Accuracy and Comprehensive Performance In terms of overall detection accuracy, ALGSP-Net achieves the optimal results on the SDD-HCS dataset. It attains a Precision of 77.46%, surpassing the baseline YOLOv11n, the high-performance Transformer-based RT-DETR, and two-stage models by margins of 5.2% and 5.5%, respectively. This significant lead evidences that the proposed defect prior and adaptive receptive field mechanisms possess superior suppression capabilities against mimetic background noise, such as water stains, shadows, and moss. Furthermore, the model secures a first-ranking F1-Score of 69.64%, indicating a stable equilibrium between recall rates and false detection errors.

Regarding localization quality, ALGSP-Net dominates across all comparative metrics, achieving an mAP50 of 72.78%, an mAP75 of 52.13%, and an mAP50-95 of 48.71%. Notably, its mAP50-95 performance marks a substantial gain of 2.18% over the 46.53% score of YOLOv13n and a 2.38% improvement over the 46.33% recorded by YOLOv12n. Even when benchmarked against the computationally intensive RT-DETR, ALGSP-Net retains a competitive edge of 0.35%. These results attest to the reliability of ALGSP-Net in bounding box regression under strict IoU thresholds, proving particularly effective for pathological targets characterized by slender cracks and irregular boundaries.

From a “Recall-Cost” perspective, although RT-DETR yields the highest Recall and near-optimal mAP50-95, its computational burden is substantial, requiring 28.46 million parameters and 100.6 GFLOPs. This high overhead renders it cost-prohibitive for edge deployment scenarios. In sharp contrast, ALGSP-Net achieves higher Precision and mAP50-95 with a mere 2.70 million parameters and 6.7 GFLOPs, demonstrating a decisive efficiency advantage.

When benchmarked against the lightweight YOLO series, YOLOv11n, v12n, and v13n operate within a similar computational range of 6.2 to 6.3 GFLOPs and a parameter size between 2.45 and 2.58 million. However, their mAP50-95 scores of 45.74%, 46.33%, and 46.53%, respectively, lag significantly behind our method. Furthermore, the computational load of ALGSP-Net is drastically lower than that of legacy models like Faster R-CNN and SSD. This confirms that the performance gains of ALGSP-Net stem primarily from structural enhancements—specifically the effective representation of fine-grained defect morphology and complex background suppression—rather than a brute-force expansion of computational scale.

#### 4.4.2. Detection Experiment Analysis on the SDD-HCS Dataset

**Figure 11:**
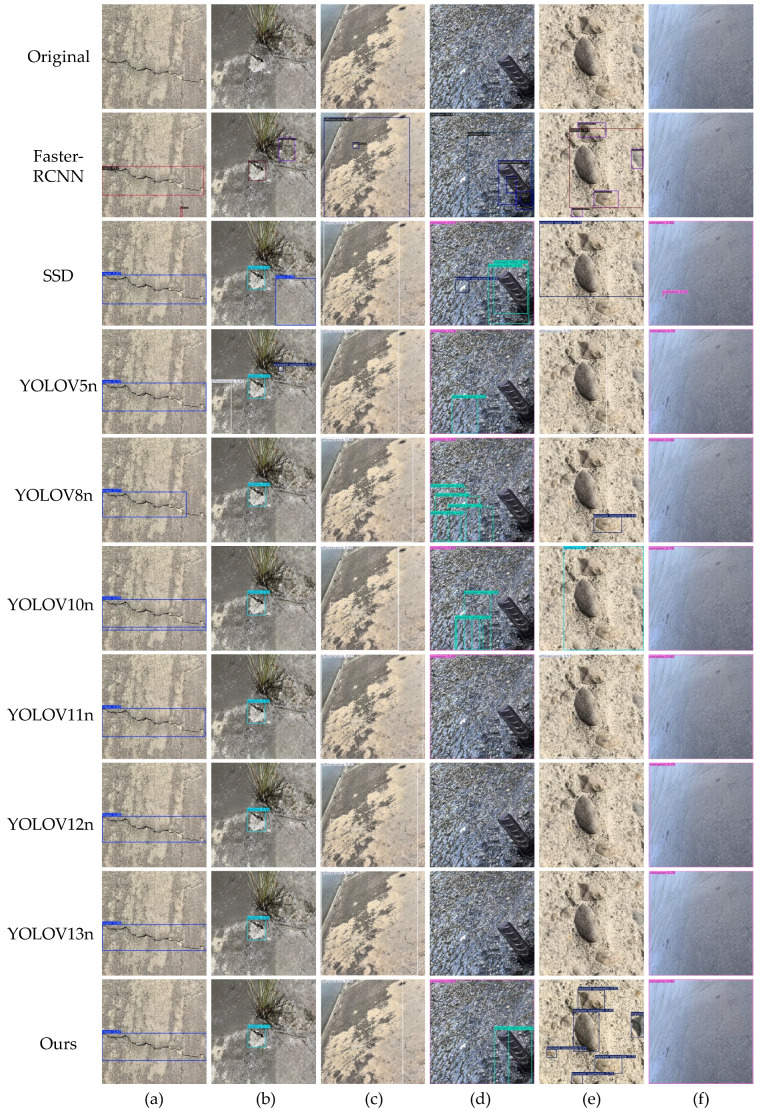
Visualization of detection results comparison on the SDD-HCS test set. Sub-figures represent the detection performance for distinct defect types: (**a**) Crack; (**b**) Spalling; (**c**) Efflorescence; (**d**) Exposed rebar; (**e**) Exposed aggregate; and (**f**) Corrosion.

To intuitively validate the robustness of ALGSP-Net under complex real-world conditions, Figure 11 presents a comparative visualization of detection results against representative state-of-the-art models across six typical defect categories. The qualitative results demonstrate that ALGSP-Net exhibits distinct advantages in addressing multifaceted challenges encompassing misclassification, missed detections, and localization drift.

For linear defects characterized by extreme aspect ratios, such as Cracks, bounding boxes generated by Faster-RCNN and SSD tend to be excessively coarse, encapsulating substantial background noise. In contrast, the predictions from ALGSP-Net adhere tightly to the trajectory and curvature of the fracture. This precision is attributed to the DaRFConv module, where deformable convolution kernels adapt to the defect’s skeletal features, ensuring complete coverage while significantly minimizing background redundancy. Similarly, in Exposed Rebar scenarios populated with gravel interference, our model accurately delineates slender rebar targets, avoiding the fragmented detections or localization deviations observed in YOLOv8n and YOLOv10n.

The detection of Exposed Aggregate presents a challenge due to the minute scale and dense distribution of targets. As illustrated in the fifth column of Figure 11, SSD and earlier YOLO iterations suffer from severe missed detections, failing to distinguish individual aggregate particles from the rough concrete surface. Conversely, leveraging the MA-C2f module for recursive aggregation of deep and shallow features, ALGSP-Net successfully preserves fine-grained textural details. This capability enables high recall rates in dense regions, distinctly identifying individual aggregate instances.

Efflorescence and Corrosion often exhibit ambiguous boundaries and are easily confused with uneven illumination or water stains. Comparative results reveal that YOLOv11n and YOLOv12n are prone to false positives when handling such indistinct boundaries, misinterpreting environmental artifacts as defects. ALGSP-Net, however, demonstrates superior semantic discrimination. By utilizing the GARF-D2GF mechanism, the model effectively suppresses unstructured environmental noise, generating high-confidence responses exclusively for genuine defect regions.

The visual evidence aligns consistently with the quantitative metrics presented in Section 4.4.1, confirming that ALGSP-Net possesses superior feature representation capabilities and noise robustness within complex hydraulic environments.

#### 4.4.3. Analysis of Single-Class Defect Detection Performance on the SDD-HCS Dataset

**Figure 12:**
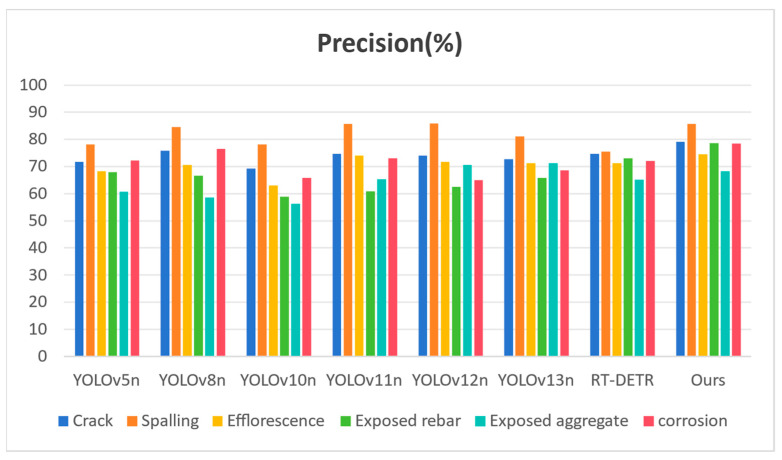
Bar chart comparing Precision metrics for different defect categories across various models.

**Figure 13:**
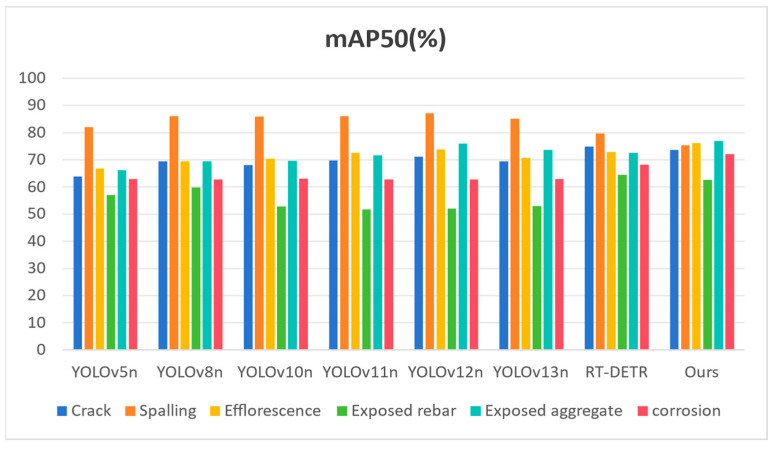
Bar chart comparing mAP50 metrics for different defect categories across various models.

To provide a granular assessment of the discriminative capabilities of ALGSP-Net across distinct defect typologies, we conducted a comparative analysis focused on six typical hydraulic concrete surface defects: crack, spalling, efflorescence, exposed rebar, exposed aggregate, and corrosion. Table 4 and Figure 12 present the Precision metrics for each model across these categories, while Table 5 and Figure 13 detail the corresponding mAP50 results.

Cracks and exposed rebar are characterized by extreme aspect ratios and complex, reticulated topological structures. The primary detection challenge lies in accurately delineating their skeletal edges without introducing background noise from the surrounding concrete. As detailed in Table 4, ALGSP-Net achieves leading Precision scores of 79.20% for cracks and 78.58% for exposed rebar, ranking first among all comparative models. Notably, in the detection of exposed rebar, our method outperforms the Transformer-based RT-DETR, which records a Precision of 72.99%, by a significant margin exceeding 5%. This performance gain provides compelling evidence for the efficacy of the DaRFConv module. By breaking the constraints of fixed sampling grids, the convolution kernels in ALGSP-Net elastically extend along the slender or curved defect skeletons. This mechanism ensures comprehensive feature coverage while minimizing the misclassification of adjacent background pixels, an advantage visually corroborated by the bar charts in Figure 12.

In the detection of corrosion and efflorescence, the visual similarity between defect regions and environmental artifacts such as water stains and moss patches frequently induces semantic confusion in YOLO-series models. Table 5 reveals the superior robustness of our method in suppressing such environmental false alarms. ALGSP-Net achieves an mAP50 of 72.09% for corrosion, surpassing the state-of-the-art YOLOv13n and YOLOv11n scores of 62.95% and 62.83%, respectively, by nearly 9%. Similarly, in efflorescence detection, it leads all competitors with an mAP50 of 76.11%. This substantial improvement is attributed to the integration of the D2GF dual-stream gating mechanism. By leveraging global contextual information to calibrate local feature responses, the network successfully distinguishes genuine pathological textures from unstructured environmental noise, effectively resolving the issue of semantic ambiguity.

For exposed aggregate, a category defined by strong granularity and irregular boundaries, ALGSP-Net secures the best mAP50 of 76.88% as shown in Table 5 while maintaining a high Precision of 68.32%. This indicates that the MA-C2f module effectively aligns deep semantic features with shallow textural details during the aggregation phase, ensuring the acute capture of these densely distributed, small-scale defects. Furthermore, in spalling detection, despite intense competition across models in mAP50 metrics, ALGSP-Net maintains an exceptionally high Precision of 85.65%, demonstrating the model’s high confidence and reliability in generating prediction boxes.

Synthesizing the Precision and mAP50 results, ALGSP-Net not only delivers superior overall metrics but, crucially, overcomes the detection bottlenecks inherent in existing mainstream models regarding “hard” classes such as corrosion and exposed rebar. These results demonstrate the practical engineering value of the model in complex hydraulic scenarios. The findings confirm that the proposed adaptive local–global synergistic perception mechanism effectively enhances the model’s adaptability to varying defect geometries and scale distributions, providing balanced and reliable performance support for multi-type defect detection in challenging hydraulic environments.

#### 4.4.4. Ablation Studies

**Figure 14:**
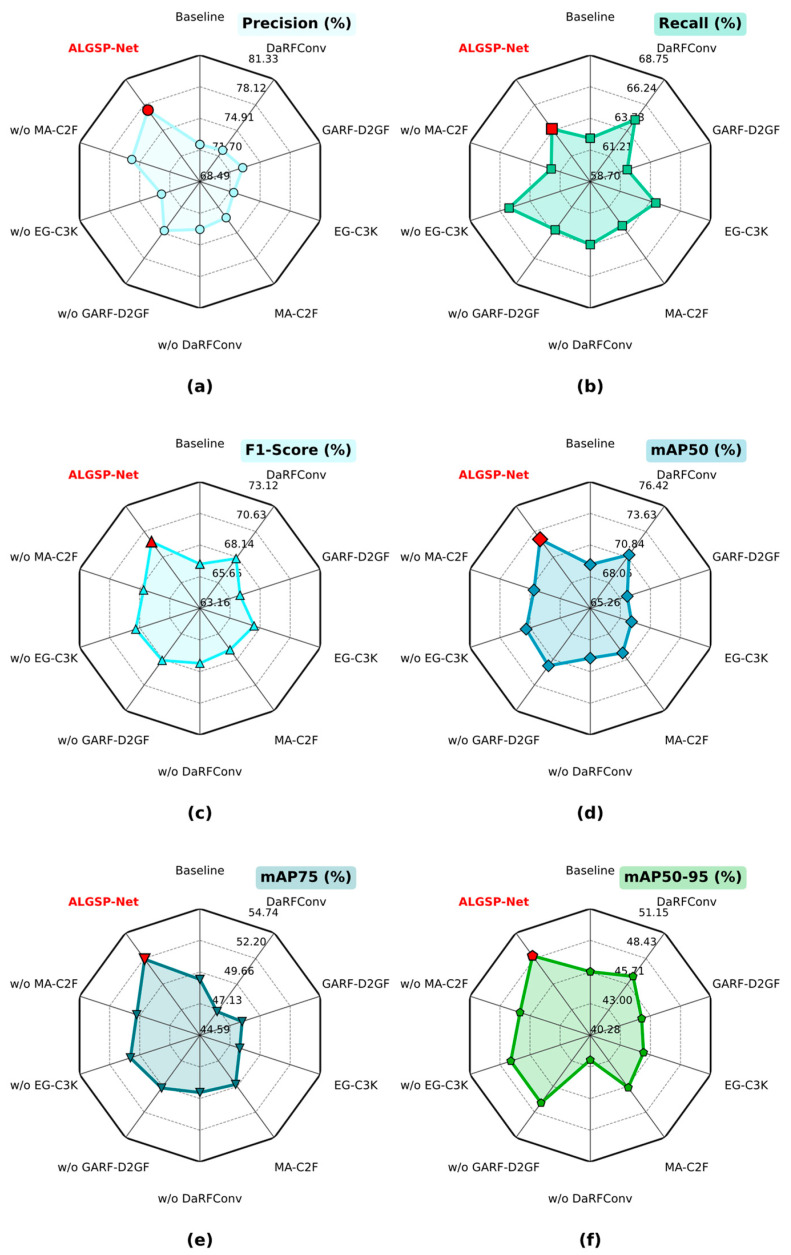
Bar chart comparing mAP50 metrics for different defect categories across various models. (**a**) Comparison of Precision; (**b**) Comparison of Recall; (**c**) Comparison of F1-Score; (**d**) Comparison of mAP50; (**e**) Comparison of mAP75; (**f**) Comparison of mAP50-95.

To systematically quantify the isolated contributions and synergistic mechanisms of the constituent innovative modules within ALGSP-Net, we designed a stepwise decoupled ablation study on the SDD-HCS dataset. Establishing the unaugmented network as the Baseline, we adopted a strategy of progressive integration to incorporate the DaRFConv, GARF-D2GF, EG-C3k, and MA-C2f modules sequentially. Table 6 tabulates the quantitative results across the dimensions of Precision, Recall, multi-threshold mAP, and computational overhead, while Figure 14 utilizes a radar chart to visually delineate the comprehensive performance envelope under each configuration.

Comparing the first and second rows of Table 6, the solitary introduction of the DaRFConv module yields a statistically significant improvement in Recall, rising from a baseline of 62.15% to 64.77%, alongside a nearly 2% growth in mAP50. This gain empirically verifies that by endowing convolution kernels with the capacity for dynamic deformation aligned with defect morphology, the model more effectively captures cracks and interlaced exposed rebar regions characterized by high topological heterogeneity. This mechanism substantially alleviates feature loss caused by fixed-grid sampling, thereby reducing the false negative rate.

When the GARF-D2GF mechanism is integrated independently, the model exhibits a marked advantage in Precision, reaching 73.06% and outperforming most single-module configurations. This indicates that by utilizing global context as a ‘semantic calibrator’, the D2GF gating mechanism precisely identifies and excises unstructured interference—such as water stains and moss—that shares textural similarities with defects. However, this suppression mechanism inherently induces a more conservative prediction strategy. When dealing with ambiguous samples where defect features are weak or heavily obscured by background textures, the gating module may classify them as noise, leading to a slight increase in false negatives. Consequently, this rigorous filtration results in a minor decrease in Recall from 62.15% to 61.79%, but the trade-off yields higher confidence in detected targets, significantly lowering the system’s false alarm rate.

The EG-C3k and MA-C2f modules focus on detail preservation within the feature propagation chain. Experimental data reveals that both modules stably enhance high-threshold localization metrics, such as mAP75 and mAP50-95, while maintaining a lightweight architecture. Notably, the inclusion of MA-C2f propels mAP50 to 70.12%, suggesting that the recursive gating aggregation strategy effectively aligns deep and shallow semantics during feature fusion, reinforcing the capture of topological details at the edges of minute defects.

To further validate the necessity of each constituent component within ALGSP-Net, we conducted a “Leave-one-out” experiment, wherein individual modules were systematically excised from the complete architecture to observe subsequent performance degradation, refer to rows 6–9 in Table 6. The results underscore the indispensable role of each module in establishing a robust detector.

Replacing DaRFConv with standard convolution precipitates a marked decline in mAP50 from 72.78% to 69.65%, accompanied by a drop in Precision to 73.34%. This performance deterioration indicates that feature extractors lacking geometric adaptability struggle to cope with the complex topologies of cracks and exposed rebar on hydraulic concrete surfaces. The fixed sampling grid of standard convolutions fails to accurately cover irregular defect regions, compromising bounding box regression quality and overall localization accuracy.

The removal of the GARF-D2GF leads to a decrease in Precision by nearly 3%, alongside a regression in mAP50 to 71.52%. This outcome confirms the centrality of GARF-D2GF in environmental noise suppression. Without global contextual calibration, the model becomes prone to misclassifying high-frequency background textures as defects, thereby escalating false positives and diminishing detection reliability.

When the EG-C3k module is omitted, the model sustains a high Recall of 65.48%; conversely, Precision drastically deteriorates to 72.59%, ranking near the bottom of all evaluated variants. This imbalance of “high recall, low precision” suggests that the EG-C3k module is critical for feature refinement. In its absence, the feature extraction process lacks effective gradient flow control, causing the model to behave aggressively in prediction; although it captures more targets, it simultaneously introduces substantial misdetections, thereby impairing the overall F1-Score.

The excision of the multi-scale aggregation module inflicts the most severe penalty on Recall and mAP50-95. The significant drop in Recall—falling even below the Baseline—demonstrates that without the recursive fusion mechanism of MA-C2f, deep semantic information cannot effectively guide shallow features, resulting in the missed detection of minute or ambiguous defects. Furthermore, the decline in high-IoU mAP metrics attests to the module’s pivotal role in achieving pixel-level feature alignment and enhancing boundary localization precision.

Upon the simultaneous integration of all four modules, ALGSP-Net achieves optimal or near-optimal performance across all metrics: Precision reaches 77.46%, Recall stands at 63.88%, F1-Score improves to 69.64%, and mAP50 attains 72.78%. As observed in the radar chart in Figure 14, the complete model forms the largest coverage area across key dimensions spanning Precision, mAP50, mAP75, and mAP50-95. This validates the distinct complementary roles of each component: DaRFConv and EG-C3k enhance geometric perception of irregular defects; GARF-D2GF suppresses false alarms in complex backgrounds; and MA-C2f plays a core role in multi-scale fusion and gradient stability. Together, they construct a closed loop of geometric enhancement, semantic calibration, and recursive aggregation, which is pivotal for ALGSP-Net to achieve high-precision and robust detection in complex hydraulic scenarios.

#### 4.4.5. Performance Evaluation on the Public Concrete Defect Dataset

**Figure 15:**
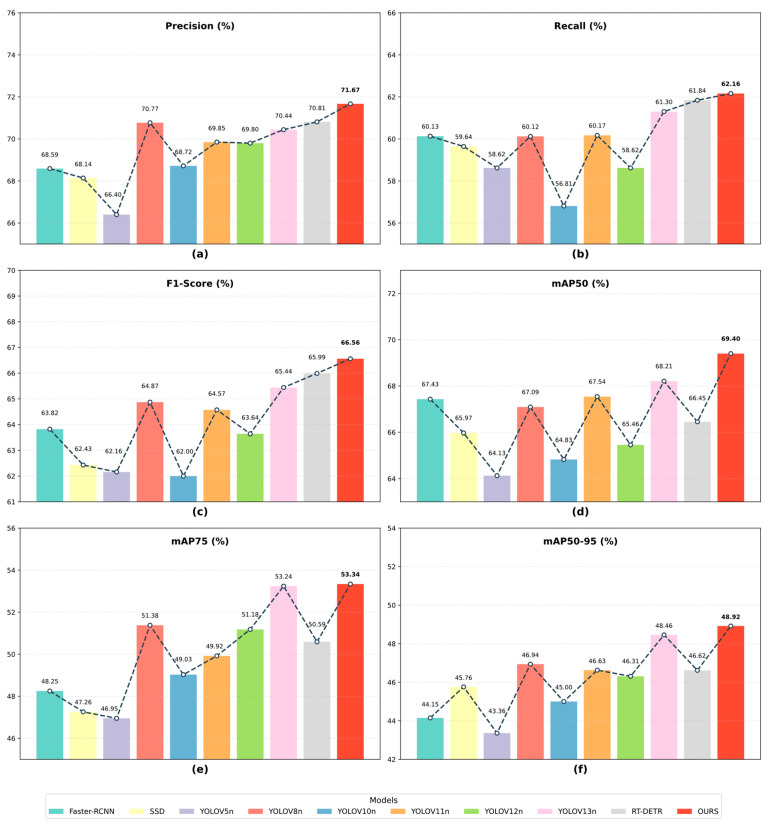
Comparative performance analysis of various detection models on the public concrete defect dataset. The dashed lines connect the metric values to visualize the performance variation across different models. (**a**) Comparison of Precision; (**b**) Comparison of Recall; (**c**) Comparison of F1-Score; (**d**) Comparison of mAP50; (**e**) Comparison of mAP75; (**f**) Comparison of mAP50-95.

To rigorously evaluate the generalization capability of ALGSP-Net across divergent data distributions and imaging environments, we extended our comparative analysis to include a publicly available concrete defect dataset. To ensure experimental consistency and comparability, we maintained an evaluation protocol identical to that of the SDD-HCS dataset, covering the same baseline models, training hyperparameters, and performance metrics. Table 7 and Figure 15 provide a comprehensive quantitative assessment, highlighting model performance under conditions where textural patterns, illumination intensities, and defect distributions differ significantly from the proprietary training domain.

Quantitative Analysis of Generalization Performance As evidenced by Table 7 and Figure 15, ALGSP-Net maintains a consistent lead across all evaluation metrics on the public dataset. The proposed model achieves a highest Precision of 71.67% and a Recall of 62.16%, culminating in a superior F1-Score of 66.56% that outperforms all comparative methods. In terms of comprehensive detection capability, our model secures a top-ranking mAP50 of 69.40%. More notably, under stricter localization requirements, ALGSP-Net attains scores of 53.34% for mAP75 and 48.92% for mAP50-95. These results significantly surpass formidable competitors, including the latest YOLOv13n and the Transformer-based RT-DETR, demonstrating that the proposed method enhances defect detection capacity generically rather than merely overfitting to specific proprietary scenarios.

Efficiency-Accuracy Trade-off Analysis From an efficiency perspective, the two-stage Faster R-CNN yields an mAP50-95 of 44.15% yet incurs a prohibitive computational cost of 90.93 GFLOPs. Similarly, while RT-DETR delivers a competitive Recall of 61.84% and F1-Score of 65.99%, its substantial resource demand, necessitating 103.5 GFLOPs and 31.996 million parameters, renders it less suitable for engineering deployment. In sharp contrast, ALGSP-Net achieves an optimal mAP50-95 of 48.92% and a peak F1-Score of 66.56% while maintaining a low computational footprint of 6.7 GFLOPs. This confirms that the architecture effectively balances high-precision detection with computational efficiency, retaining its practical advantage even on external public datasets.

## 5. Conclusions

This study developed the ALGSP-Net framework and the SDD-HCS dataset to address the persistent challenges of topological heterogeneity and environmental noise in hydraulic concrete defect detection. By integrating DaRFAConv and ARFConv modules, the method overcomes the limitations of fixed grid sampling, enabling adaptive perception of irregular defect morphologies such as slender cracks. Furthermore, the proposed Global-Local Synergistic mechanism effectively utilizes global context to calibrate local responses, mitigating false alarms caused by background interference like water stains. Experimental results on the SDD-HCS dataset confirm that the method achieves an mAP50 of 72.78%, demonstrating superior precision and robustness across six typical defect categories compared to existing baselines.

These findings provide a viable technological solution for the automated inspection and maintenance of hydraulic infrastructure. Future research will focus on two primary directions: (1) implementing model lightweighting techniques, such as pruning and knowledge distillation, to facilitate real-time edge deployment on UAV platforms; and (2) exploring multi-modal data fusion to further enhance detection reliability under extreme conditions, such as low-light or turbid environments.

## Figures and Tables

**Table 1:** Hardware and software specifications of the experimental environment.

Component	Specification
CPU	Intel Core i914000K
GPU	NVIDIA RTX 4090 (24 GB)
RAM	64 GB
Operating System	Ubuntu 24.04
Programming Language	Python 3.12
Deep Learning Framework	Pytorch 2.4.1
CUDA Version	12.4

**Table 2:** The critical hyperparameters employed during the training process.

Hyperparameters	Epoch	Batch	Lr0/f	Momentum	Imgsz	Optimizer
Value	300	32	0.01	0.973	640 × 640	SDG

**Table 3:** Comparison of key performance metrics across different models on the SDD-HCS dataset.

Model	Precision (%)	Recall (%)	F1-Score (%)	mAP50 (%)	mAP75 (%)	mAP50-95 (%)	Params (B)	GFLOPS (G)
Faster-RCNN	69.88	61.32	64.57	68.68	48.79	44.67	41,374,253	90.9
SSD	68.34	62.43	67.52	67.15	48.04	43.86	24,414,547	30.7
yoloV5n	69.86	61.36	64.94	66.43	44.40	41.89	2,504,114	7.1
yoloV8n	72.12	64.11	67.30	69.50	49.32	45.85	3,006,818	8.1
yoloV10n	65.20	65.34	64.96	68.34	48.68	44.85	2,266,338	6.5
yoloV11n	72.26	62.15	66.64	69.11	49.07	45.74	2,583,322	6.3
yoloV12n	71.65	64.59	67.81	70.61	50.86	46.33	2,557,898	6.3
yoloV13n	71.91	63.33	67.25	69.15	50.10	46.53	2,449,065	6.2
RT-DETR	71.96	65.98	68.60	72.14	51.74	48.36	28,455,590	100.6
OURS	77.46	63.88	69.64	72.78	52.13	48.71	2,698,761	6.7

**Table 4:** Comparison of Precision metrics for different defect categories across various models.

Defect	YOLOv5n (%)	YOLOv8n (%)	YOLOv10n (%)	YOLOv11n (%)	YOLOv12n (%)	YOLOv13n (%)	RT-DETR (%)	Ours (%)
Crack	71.81	75.84	69.20	74.62	74.03	72.71	74.75	79.20
Spalling	78.07	84.52	78.21	85.64	85.92	81.07	75.56	85.65
Efflorescence	68.29	70.59	62.94	74.09	71.67	71.29	71.28	74.59
Exposed rebar	68.01	66.71	58.87	60.89	62.59	65.81	72.99	78.58
Exposed aggregate	60.77	58.52	56.22	65.33	70.65	71.27	65.08	68.32
Corrosion	72.21	76.55	65.79	73.02	65.02	68.62	72.10	78.44

**Table 5:** Comparison of mAP50 metrics for different defect categories across various models.

Defect	YOLOv5n (%)	YOLOv8n (%)	YOLOv10n (%)	YOLOv11n (%)	YOLOv12n (%)	YOLOv13n (%)	RT-DETR (%)	Ours (%)
Crack	63.8	69.51	68.04	69.82	71.22	69.38	74.85	73.62
Spalling	81.95	86.02	85.95	86.09	87.14	85.20	79.74	75.36
Efflorescence	66.78	69.43	70.32	72.52	73.81	70.72	72.94	76.11
Exposed rebar	56.97	59.85	52.91	51.78	52.01	53.05	64.46	62.61
Exposed aggregate	66.15	69.45	69.68	71.61	75.95	73.65	72.57	76.88
Corrosion	62.91	62.74	63.14	62.83	62.74	62.95	68.28	72.09

**Table 6:** Results of ablation experiments on the SDD-HCS dataset.

Defect	Precision (%)	Recall (%)	F1-Score (%)	mAP50 (%)	mAP75 (%)	mAP50-95 (%)	Params (B)	GFLOPS (G)
Baseline	72.26	62.15	66.64	69.11	49.07	45.74	2,583,322	6.3
+DaRFConv	72.43	64.77	68.00	71.09	46.94	46.53	2,625,990	6.5
+GARF-D2GF	73.06	61.79	66.48	68.69	48.15	44.92	2,767,861	6.4
+EG-C3k	72.10	64.18	67.64	69.09	47.95	45.09	2,524,794	6.2
+MA-C2f	73.02	63.03	67.19	70.12	49.46	45.83	2,655,242	6.4
w/o DaRFConv	73.34	63.70	67.48	69.65	49.18	42.40	2,514,222	6.6
w/o GARF-D2GF	74.64	63.43	68.22	71.52	49.82	47.46	2,514,222	6.6
w/o EG-C3k	72.59	65.48	68.47	71.20	50.45	47.45	2,759,529	6.8
w/o MA-C2f	75.76	61.96	67.82	70.47	49.92	46.62	2,628,416	6.6
ALGSP-Net	77.46	63.88	69.64	72.78	52.13	48.71	2,698,761	6.7

**Table 7:** Comparison of key performance metrics across different models on the public concrete defect dataset.

Model	Precision (%)	Recall (%)	F1-Score (%)	mAP50 (%)	mAP75 (%)	mAP50-95 (%)	Params (B)	GFLOPS (G)
Faster-RCNN	68.59	60.13	63.82	67.43	48.25	44.15	41,374,253	90.9
SSD	68.14	59.64	62.43	65.97	47.26	45.76	24,414,547	30.7
yoloV5n	66.4	58.62	62.16	64.13	46.95	43.36	2,504,114	7.1
yoloV8n	70.77	60.12	64.87	67.09	51.38	46.94	3,006,818	8.1
yoloV10n	68.72	56.81	62.00	64.83	49.03	45.00	2,266,338	6.5
yoloV11n	69.85	60.17	64.57	67.54	49.92	46.63	2,583,322	6.3
yoloV12n	69.8	58.62	63.64	65.46	51.18	46.31	2,557,898	6.3
yoloV13n	70.44	61.30	65.44	68.21	53.24	48.46	2,449,065	6.2
RT-DETR	70.81	61.84	65.99	66.45	50.59	46.62	31,996,070	103.5
OURS	71.67	62.16	66.56	69.4	53.34	48.92	2,698,761	6.7

## Data Availability

The raw data supporting the conclusions of this article will be made available by the authors on request.

## Abbreviations

The following abbreviations are used in this manuscript:

ALGSP-Net	Adaptive Local–Global Synergistic Perception Network
SDD-HCS	Surface Defect Dataset for Hydraulic Concrete Structures
DaRFAConv	Defect-aware Receptive Field Aggregation Convolution
ARFConv	Adaptive Dynamic Receptive Field Convolution
GARFConv	Global-Local Synergistic Receptive Field Aggregation Convolution
D2GF	Defect-aware Dual-stream Gating Fusion
CNNs	Convolutional Neural Networks
EG-C3k	Elastic Geometric Encoding
MA-C2f	Morphology-Adaptive Recursive Aggregator
YOLO	You Only Look Once
LSKA	Large Selective Kernel Attention
ViT	Vision Transformers
GAP	Global Average Pooling
MLP	Multilayer Perceptron
UAV	Unmanned Aerial Vehicle
mAP	mean Average Precision
IoU	Intersection over Union
FPS	Frames Per Second
FMOE	ourier-Mixture of Experts
SMM	State Space Models
CSP	Cross Stage Partial
DCNs	Deformable Convolution Networks
DWConv	Depthwise Convolution
ReLU	Rectified Linear Unit
BN	Batch Normalization
DFL	Distribution Focal Loss
BCE	Binary Cross Entropy
CIoU	Complete Intersection over Union
